# Growth and single cell kinetics of the loricate choanoflagellate *Diaphanoeca grandis*

**DOI:** 10.1038/s41598-019-50998-0

**Published:** 2019-10-10

**Authors:** Niels Thomas Eriksen, Jakob Tophøj, Rasmus Dam Wollenberg, Teis Esben Sondergaard, Peter Funch, Per Andersen

**Affiliations:** 1 Department of Chemistry and Bioscience, Aalborg University, Fredrik Bajers Vej 7H, DK-9220 Aalborg, Denmark; 2 Department of Bioscience, Aarhus University, Ny Munkegade 116, DK-8000 Aarhus, Denmark

**Keywords:** Water microbiology, Marine biology

## Abstract

Choanoflagellates are common members of planktonic communities. Some have complex life histories that involve transitions between multiple cell stages. We have grown the loricate choanoflagellate *Diaphanoeca grandis* on the bacterium *Pantoea* sp. and integrated kinetic observations at the culture level and at the single cell level. The life history of *D. grandis* includes a cell division cycle with a number of recognisable cell stages. Mature, loricate *D. grandis* were immobile and settled on the bottom substratum. Daughter cells were ejected from the lorica 30 min. after cell division, became motile and glided on the bottom substratum until they assembled a lorica. Single cell kinetics could explain overall growth kinetics in *D. grandis* cultures. The specific growth rate was 0.72 day^−1^ during exponential growth while mature *D. grandis* produced daughter cells at a rate of 0.9 day^−1^. Daughter cells took about 1.2 h to mature. *D. grandis* was able to abandon and replace its lorica, an event that delayed daughter cell formation by more than 2 days. The frequency of daughter cell formation varied considerably among individuals and single cell kinetics demonstrated an extensive degree of heterogeneity in *D. grandis* cultures, also when growth appeared to be balanced.

## Introduction

*Diaphanoeca grandis* is a marine/brackish water solitary loricate choanoflagellate with morphotypes distributed globally^1^. It has been observed year-round in temperate waters^2–4^ mostly suspended in the water column^2^ and most frequently near the coast^4^. Choanoflagellates are filter feeders and feed primarily on picoplankton such as smaller bacterial cells^5–7^. Along with other heterotrophic nanoflagellates these organisms play important roles in marine environments where they depress the concentration of bacteria in the water column^8,9^ and mediate the transfer of bacterial biomass to higher trophic levels of aquatic food webs^10^.

**Figure 1:**
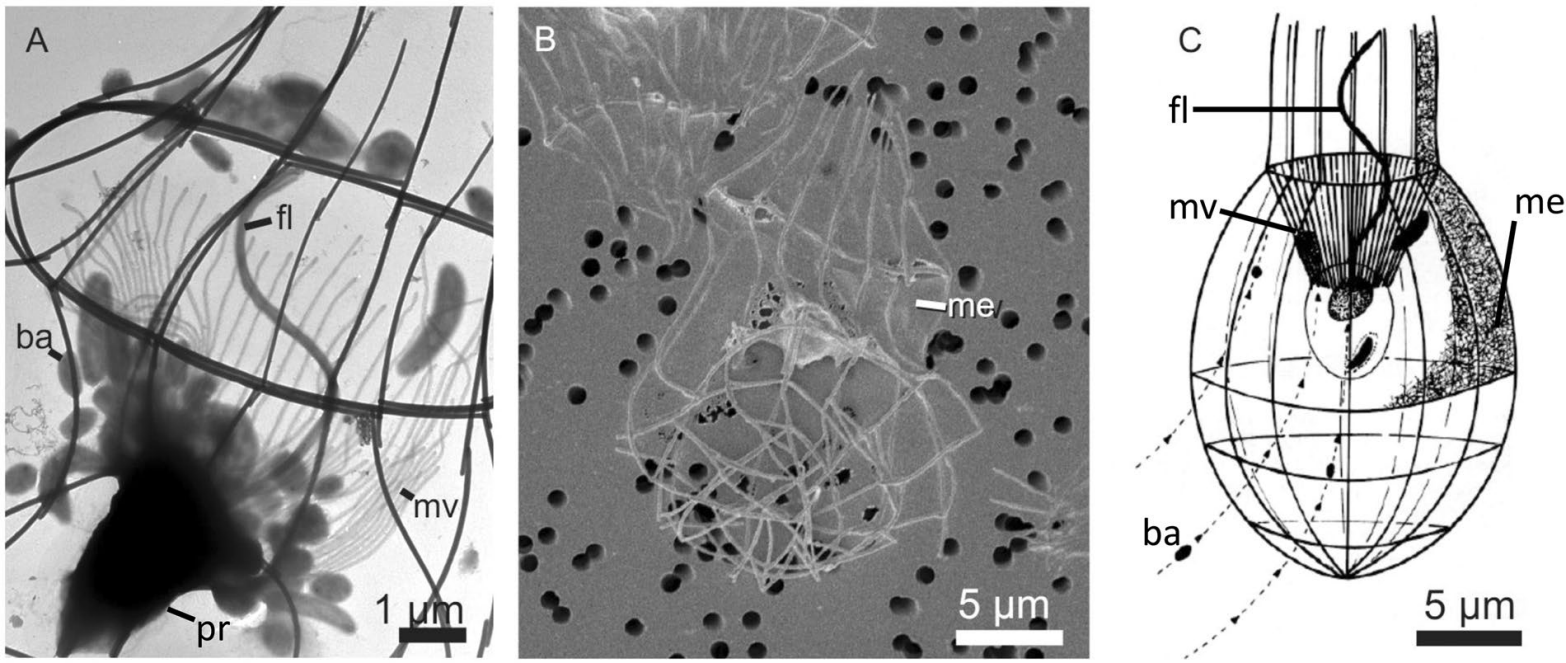
*Diaphanoeca grandis*. (**A**) Wholemount (TEM), ba, bacteria; fl, flagellum; mv, microvilli collar; pr, protoplast. Fluid flow direction from lower left corner towards upper right corner. (**B**) Lorica with apical membranous veil (me) concealing collar and flagellum (SEM, fixed on 1 μm polycarbonate filter; hexamethyldisilazane dried). (**C**) (redrawn from Andersen^3^). Schematic drawing with flow lines (arrows) and transport of bacteria (black dots) to the microvilli collar.

*D. grandis* belongs to the family Stephanoecidae and has characteristic choanoflagellate morphology (Fig. 1). The protoplast measures 4–6 μm and possesses a 9 μm long apical flagellum surrounded by a collar of approximately 50 microvilli^3,11^. The surrounding lorica measures approximately 16 × 28 μm and is built from approximately 120 silicate sticks (costal strips). The collar anchors the protoplast within the lorica. Part of the lorica is covered by a thin membranous veil consisting of a meshwork of microfibrils embedded in a matrix^3,12^. The flagellum generates a water flow through the collar where bacteria and other particles are captured (Fig. 1A). Water exits via the part of the lorica named the chimney^3,13^. In Stephanoecidae, the costal strips that are to be used by daughter cells to construct new lorica are synthesised by the parental protoplast prior to cell division and transported on the surface of the daughter cell when it leaves the parental lorica^11,14^. The specific growth rate of *D. grandis*^3^ and the related species, *Stephanoeca diplocostata*^15^ follows Monod type kinetics with suspended bacteria as the growth limiting food source. Cultivation of *D. grandis* have shown a maximum specific growth rate, *μ*_*max*_ = 0.12 h^−1^ at 15 °C, a half-saturation constant, *K*_*b*_ = 2,400 bacterial cells (*Pseudomonas* sp.) per μL, and a maximal ingestion rate of approximately 40 *Pseudomonas* per h^3,16^.

**Figure 2:**
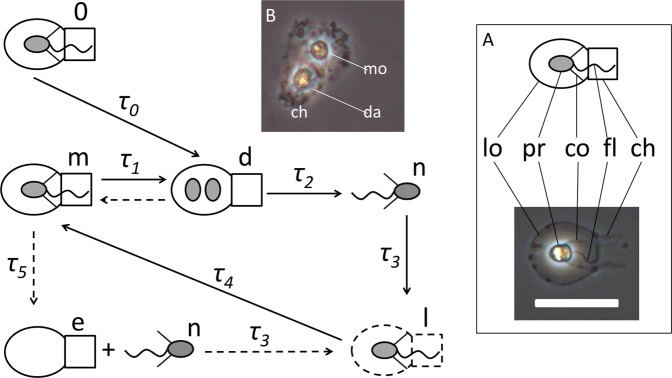
*Diaphanoeca grandis* life history. *D. grandis* added in inoculum until it has passed through first cell division, 0. Mature, loricate *D. grandis*, m. Dividing protoplast, d. Motile, non-loricate cell, n. Cell assembling new lorica, l. Empty lorica, e. Solid arrows indicate transitions between cell stages in the cell division cycle. Characteristic life times of different cell stages: Time until cells divide the first time, *τ*_0_. Time between cell divisions, *τ*_1_. Life time of stage with two protoplasts (mother and daughter) inside the lorica, *τ*_2_. Life time of motile, non-loricate cell stage, *τ*_3_. Time expenditure for assembly of new lorica, *τ*_4_. Dashed lines indicate protoplast abandoning its lorica and forming a motile cell that subsequently constructs a new lorica. Time span from protoplast went through its final cell division until it abandons its lorica, *τ*_5_. Inset A: Cell components of loricate *D. grandis*. Lorica, lo. Protoplast, pr. Collar, co. Flagellum, fl. Chimney, ch. Scale bar = 20 μm. Inset B. Dividing protoplast. Mother, mo, remains in parental lorica. Daughter, da, about to exit via the chimney, ch.

The life history of *D. grandis* is shown in Fig. 2. The cell division cycle proceeds via a series of recognizable cell stages^11^. The specific growth rate will depend on the life time of each of the different cell stages. Cell division takes place within the parental lorica at intervals, *τ*_1_. Two protoplasts will then be present within the parental lorica for a period of time, *τ*_2_. Subsequently, one protoplast, named the daughter protoplast, leaves the parental lorica via the chimney (Fig. 2, Inset B) and spends a period of time, *τ*_3_ as a motile, non-loricate cell. The second protoplast, here named the mother protoplast, remains inside the parental lorica. Finally, the daughter cell spends a period of time, *τ*_4_ assembling a new lorica before it has matured. The protoplast may also leave its lorica after a time, *τ*_5_ and become motile for a period of time before it again assembles a new lorica. Microbial batch cultures often show an initial lag phase during which the cells adjust to novel conditions before growth commences. In Fig. 2, the time it takes from a cell is added with the inoculum and until it undergoes its first cell division and thereafter is recognized as a mature, loricate choanoflagellate is denoted *τ*_0_.

Choanoflagellates can be solitary as well as colonial and is the sister group to metazoans^17^. The two aloricate species, *Monosiga brevicollis* and *Salpingoeca rosetta* have been suggested as model organisms for the study of early animal evolution^18,19^. Both species have life histories that include several morphologically different cell stages^18,20,21^ and belong to the choanoflagellate order, Craspedida^22^. The loricate choanoflagellates belong to the second choanoflagellate order, Acanthoecida^22^. These may also have life histories that include more cell stages (Fig. 2) but only few studies have focussed on their growth kinetics, presumably because loricate choanoflagellates are difficult to bring into culture. A few species, including *D. grandis* have however been characterised from cultivation studies^3,11,23^. The purpose of this study is to integrate kinetic observations at the culture level with the kinetics of the different steps in the cell division cycle measured at the single cell level in order to develop a structured kinetic growth model for loricate choanoflagellates. Such an approach may deepen our understanding of the life history of *D. grandis* and qualify the interpretation of kinetic data from cultures of loricate choanoflagellates.

## Methods and Materials

### Sampling and isolation of *Diaphanoeca grandis* and *Pantoea* sp

*Diaphanoeca grandis*^24^ was isolated from a 5 L surface water sample April 10, 2016 from Mariager Fjord, Denmark (56°39′15.51″N 9°58′57.26″E) by the dilution culture method^3^. Serial dilution of enrichment cultures into autoclaved seawater (salinity = 10‰, temp. = 15 °C) containing wheat grains as feed for bacterial prey resulted in cultures that appeared to be free from other eukaryotic contaminants. These cultures were subsequently diluted in sterile seawater until a *D. grandis* concentration of approximately 1 μL^−1^ and transferred into a microtiter plate well (1 μL per well) containing 200 μL of sterile seawater enriched by 0.01 g L^−1^ of yeast extract as substrate for the bacterial population still present in the culture. After 1 week of incubation at 15 °C, each well was examined under the microscope. One isolate was selected for further studies and at 2–3 week intervals, this *D. grandis* isolate was transferred into new batch cultures at 15 °C in 10‰ autoclaved seawater supplemented by 0.01 g L^−1^ of yeast extract and named *D. grandis* MF2016-1. Other eukaryotes but *D. grandis* were not observed under the microscope.

A bacterium to be used as feed was isolated from a *Diaphanoeca grandis* wheat grain culture. First, the culture was diluted in sterile sea water until a bacterial concentration of 10 μL^−1^. Then, 20 μL was spread onto petri dishes (approximately 200 cells per petri dish) containing seawater autoclaved with 50 g L^−1^ of wheat grains. The medium was solidified by 10 g L^−1^ agar. The plates were incubated at 15 °C for 1 week, and individual bacterial colonies were isolated and transferred to sterile seawater enriched with 1 g L^−1^ of yeast extract. One bacterial isolate consisting of spherical or ellipsoid cells with a diameter of approximately 1 μm and a cell volume of approximately 0.7 μm^3^ (later identified as *Pantoea* sp.) grew as solitary, non-motile cells and supported growth of *D. grandis*. This bacterium was grown in batch cultures in an orbital shaking incubator at 15 °C until stationary phase was reached, where after the culture was harvested by centrifugation, re-suspended in sterile seawater, and used as sole prey for *D. grandis* cultures. This procedure minimized potential co-transfer of left-over components from the bacterial growth medium^25^.

*Diaphanoeca grandis* was identified based on its characteristic morphology combined with blastN analysis against the non-redundant database at NCBI^26^ after PCR amplification and partial sequencing of the silica transporter gene using the primer pair for *D. grandis*^27^. *Pantoea* sp. was identified by PCR amplification and partial sequencing followed by blastN analysis of the 16 S rDNA gene using the primer pair 27 F/1492 R for *Pantooea* sp.^28^. Experimental details were as described by Tophøj *et al*.^25^.

### Population growth and grazing in batch cultures of *Diaphanoeca grandis*

Prior to growth and grazing experiments, *Diaphanoeca grandis* inocula were precultured on *Pantoea* sp. and 1–2 mL of these precultures were used to inoculate new *D. grandis* cultures at cell concentrations of 2–10 μL^−1^ in 50 mL of sterile seawater. *Pantoea* sp. was added at cell concentrations of 4,000–26,000 μL^−1^ as the sole feed. In addition were 50–100 unidentified bacteria μL^−1^ carried over with the inocula since 1,000–2,000 bacteria μL^−1^ remained in the precultures. The *D. grandis* cultures were grown in 100 mL conical glass flasks incubated at 15 °C with no stirring. Only when samples were taken 1–2 times per day, cultures were gently shaken to form a homogeneous suspension. Attempts were also made to grow *D. grandis* in suspension cultures stirred by an orbital shaker, a planktonic wheel, or by gentle sparging.

*D. grandis* and *Pantoea* sp. were simultaneously counted by flow cytometry in samples taken from the cultures. Cells were stained by addition of 1 μL acridine orange (Sigma-Aldrich) solution (1 mg L^−1^) to 1 mL of culture sample. After 5 min of incubation, cells were counted on a Cyflow Space flow cytometer (Sysmex Partec, Germany) equipped with a 480 nm blue laser. Concentrations of *D. grandis*, *c*_*f*_ and *Pantoea* sp., *c*_*b*_ were calculated from absolute counts of particles in 0.7 mL sample volume giving rise to simultaneous forward scatter, side scatter, and green fluorescence signals. *D. grandis* concentrations were also determined from microscopically counts in a 0.0025 mm^3^ hemocytometer (Thoma, Germany).

To identify the range of bacterial concentrations where *D. grandis* grow optimally, the specific growth rate, *μ* as function of bacterial concentration *c*_*b*_, was described by

1 $$\mu ={\mu }_{{\max }}(\frac{{c}_{b}}{{K}_{b}+{c}_{b}})(1-\frac{1}{1+{e}^{(E{C}_{50}-{c}_{b})\cdot H}})$$

that combines the Monod model with a sigmoidal dose-response curve (supersaturating bacterial concentrations may reduce the filtration rate of *D. grandis*^3^ and potentially also affect growth), where *μ*_*max*_ is the maximal specific growth rate and *K*_*b*_ is a half-saturation constant. *EC*_50_ is the effective bacterial concentration at which the specific growth rate is reduced to half its maximal value. The coefficient *H* defines the steepness of the slope of the curve (additional details in Supplementary Information).

### Single cell growth kinetics

Growth of *Diaphanoeca grandis* was also monitored at the single-cell level in the oCelloScope optical detection system (Biosense Solutions Aps, Denmark), which is an automatic digital microscope equipped with a 4x optical magnification (200x effective magnification)^29,30^. In the oCelloScope, *D. grandis* was grown in NUNC^TM^ Edge 96-well plates (Thermo Scientific, USA) containing 200 μL of sterilised seawater (salinity = 10‰, temp. = 15 °C) and with *Pantoea* sp. as sole prey. To minimize evaporation, 8 mL of water was added to the surrounding plate-reservoir. *D. grandis* inocula and *Pantoea* sp. used as feed had been treated as described above, before being loaded into the microtiter plate (5 *D. grandis* μL^−1^ and 3,000–12,000 *Pantoea* sp. μL^−1^). After 1 hour of incubation to allow for sedimentation, focus and light intensity were manually adjusted for each well. Each scan at 1 min. interval (6 days total incubation) consisted of 15 separate raw images (with an image distance of 11.44 µM) and from these in-focus planes were automatically concatenated to form a series of images covering a 1,300 μm × 1,200 μm area. To preserve image quality, each frame was manually exported and subsequently imported into Fiji ImageJ ver. 2.0.0. The image stacks could be viewed as a film and allowed continuous surveillance of individual *D. grandis* for up to 6 days with recordings of several consecutive cell division cycles. From the image stacks, the characteristic life time, *τ*_0_ - *τ*_5_ of the different cell stages of individual *D. grandis* (Fig. 2) were determined.

The cell division cycle of *D. grandis* depicted in Fig. 2 was translated into a structured kinetic growth model, describing the number of cells, *N* in batch cultures of *D. grandis*

2 $$\frac{d{N}_{0}}{dt}=-\,{k}_{0}{N}_{0}$$

3 $$\frac{d{N}_{m}}{dt}={k}_{0}{N}_{0}+{k}_{4}{N}_{l}$$

4 $$\frac{d{N}_{d}}{dt}={k}_{0}{N}_{0}+{k}_{1}\frac{{c}_{b}}{{K}_{b}+{c}_{b}}{N}_{m}-{k}_{2}{N}_{d}$$

5 $$\frac{d{N}_{n}}{dt}={k}_{2}{N}_{d}-{k}_{3}{N}_{n}$$

6 $$\frac{d{N}_{l}}{dt}={k}_{3}{N}_{n}-{k}_{4}{N}_{l}$$

where numbers of each recognisable cell stage are denoted the following way; *N*_0_, loricate *D. grandis* inoculated into the culture that have not yet gone through a cell division. *N*_*m*_, mature, loricate *D. grandis*. *N*_*d*_, parental lorica with two protoplasts inside. *N*_*n*_, motile, non-loricate cell. *N*_*l*_, cell being in the process of assembling a lorica. Each term in Eqs. 2–6 represent a transition between cell stages, represented by a solid arrow in Fig. 2. Each rate constant, *k*_0_, *k*_1_, *k*_2_, *k*_3_, and *k*_4_ correspond to the inverse value of the characteristic life time of the particular cell stage in the cell division cycle, *τ*_0_^−1^, *τ*_1_^−1^, *τ*_2_^−1^, *τ*_3_^−1^, and *τ*_4_^−1^ (Fig. 2), respectively. If bacterial concentrations, *c*_*b*_ are well below *EC*_50_, the rate of daughter cell formation is assumed to follow Monod type kinetics^3^ and the rate constant, *k*_1_ must therefore be determined during the exponential growth phase.

Equations 2–6 can be modified to describe temporal changes in the concentration of *D. grandis* if cell numbers are divided by the culture volume. If the bacterial prey does not grow and growth of *D. grandis* is balanced, the bacterial concentration, *c*_*b*_ will decrease at rates described by

7 $$\frac{d{c}_{b}}{dt}=\frac{1}{{Y}_{f/b}\cdot V}\cdot \frac{dN}{dt}={Y}_{f/b}^{-1}\cdot \frac{d{c}_{f}}{dt}$$

where *Y*_*f/b*_ is the yield of flagellate cells (*D. grandis*) feasting on bacterial prey, *V* is culture volume, and *c*_*f*_ is the concentration of flagellate cells (*D. grandis*). The ingestion rate, *I* can be estimated as

8 $$I={Y}_{f/b}^{-1}\cdot \mu $$

### Linking single cell growth kinetics to population growth

Concentrations of *D. grandis* and *Pantoea* sp. in 50 mL batch cultures were simulated by the growth model, Eqs. 2–7 while the rate constants were obtained from examination of single cells in 200 μL cultures. The individual equations in the model were solved numerically using Euler’s solution at time intervals, *Δt* = 0.2 h, starting at *t* = 24–36 h (corresponding to the length of lag phases), as shown in Supplementary Information, Eqs S4–S9.

## Results

### Growth of *Diaphanoeca grandis* in batch culture

**Figure 3:**
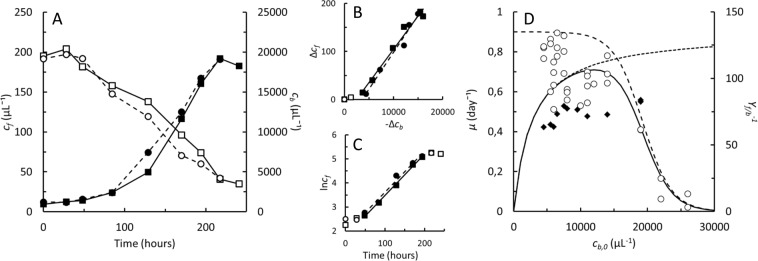
*Diaphanoeca grandis*. Two replicate batch cultures grown on *Pantoea* sp. (**A**). Concentrations of *D. grandis*, *c*_*f*_ (●, ■) and bacterial prey *Pantoea* sp., *c*_*b*_ (○, □). (**B**). Increase in concentration of *D. grandis* vs. decrease in concentration of *Pantoea* sp. Slopes of regression lines correspond to the yield of choanoflagellates (*D. grandis*) per bacterium, *Y*_*f/b*_. (**C**). ln(*c*_*D. grandis*_) vs. time, exponential growth phase indicated by solid symbols, slope of regression line equals specific growth rates. (**D**). Specific growth rates, *µ* (○) of 34 *D. grandis* cultures grown on initial *Pantoea* sp. concentrations of 4,000–26,000 μL^−1^. Solid line shows best fit of Eq. 1, *μ*_*max*_ = 0.9 day^−1^, *K*_*b*_ = 2,400 μL^−1^, *EC*_50_ = 19,000 μL^−1^, *H* = 0.46. Dotted line shows the Monod model (Supplementary Information, Eq. S1), *μ*_*max*_ = 0.9 day^−1^, *K*_*b*_ = 2,400 μL^−1^. Dashed line shows fit of a dose-response curve (Supplementary Information, Eq. S2), *μ*_*max*_ = 0.9 day^−1^, *EC*_50_ = 19,000 μL^−1^, *H* = 0.46. Reciprocal yields, *Y*_*f/b*_^*−*1^, corresponding to the number of *Pantoea* sp. consumed per *D. grandis* produced during exponential growth phase (◆).

*Diaphanoeca grandis* from Mariager Fjord was recognized from the size and shape of the lorica (Figs 1 and 2). SEM images furthermore disclosed the presence of the apical membranous veil (Fig. 1B), and the silica transporter gene (Genbank ID MN369736) showed 98% sequence identity to GenBank ID HE981738.1, *Diaphanoeca grandis*. The isolate was grown in batch culture using *Pantoea* sp. (the 16 S rDNA sequence, Genbank ID MN250297 showed 99.7% sequence identity to GenBank ID KX891536.1, *Pantoea eucalypti*) as the sole prey. All attempts to grow *D. grandis* in stirred suspension cultures were unsuccessful. *D. grandis* was therefore grown in non-stirred batch cultures on initial *Pantoea* sp. concentrations of 4,000–26,000 μL^−1^. *D. grandis*, counted by flow cytometry after resuspension of the cells, grew to maximal concentrations of approximately 200 μL^−1^. Growth was always accompanied by a decrease in the concentration of bacteria (Fig. 3A and Supplementary Information, Fig. S2). During the exponential growth phase, the average yield of *D. grandis* per bacterium, *Y*_*f/b*_ = 0.014 ± 0.001 (*n* = 27), meaning the production of one *D. grandis* occurred at the expense of *Y*_*f/b*_^*−1*^ = 74 ± 7 *Pantoea* sp. (Fig. 3B). Addition of sodium metasilicate to the seawater did not affect final concentrations of *D. grandis*. Length, diameter and volume of the protoplasts were 4.9 ± 0.7 μm, 3.9 ± 0.7 μm, and 60 ± 28 μm^3^, *n* = 259, respectively. There were considerable variability between individuals and the average protoplast volume decreased from an initial average of 95 μm^3^ to 44 μm^3^ as batch cultures progressed (Supplementary Information, Fig. S3).

Specific growth rates were determined taking into account the initial lag phase of *D. grandis* (Fig. 3C). Figure 3D shows specific growth rates measured in 30 *D. grandis* cultures grown on *Pantoea* sp. The maximal specific growth rate estimated according to the Monod model (Supplementary Information, Eq. S1), *μ*_*max*_ = 0.72 ± 0.10 day^−1^ (*n* = 24) was observed in cultures with initial *Pantoea* sp. concentrations of 4,000–15,000 μL^−1^. At higher initial *Pantoea* sp. concentrations, specific growth rate decreased. Best fit of a dose-response curve (Supplementary Information, Eq. S2) to the measured specific growth rates indicated that *EC*_50_ ≈ 19,000 bacterial cells per μL. The initial bacterial concentrations, *c*_*b,0*_ were used as rough estimates of the actual bacterial concentrations in the cultures, *c*_*b*_. Figure 3D also shows the best fit of Eq. 1 to the measured specific growth rates, using *K*_*b*_ = 2,400 bacteria per μL^3^. During exponential growth phases, the average ingestion rate was 53 *Pantoea* sp. day^−1^ (Eq. 8).

### Qualitative observations on individual *Diaphanoeca grandis*

When *Diaphanoeca grandis* was grown in 96-well plates, individuals were continuously observed in the oCelloScope for periods of up to 6 days. *D. grandis* settled on the bottom of the wells within 2–3 h. Loricate cells, resting on the bottom, were immobile. All *D. grandis* present in the filmed area on Day 0 could therefore be followed until the experiment was terminated on Day 6 (Supplementary Information, Figs. S4-S5). Protoplast and lorica were visible in all individuals. In a few individuals, also the beating flagellum could be seen. The elongated cytoplasmic strand used to move daughter cells out of the parental lorica^14^ was occasionally visible during the cell separation phase (Supplementary Information, Figs. S8 and S11 and Supplementary Video S1). Most *D. grandis* settled with their longitudinal axis in parallel to the bottom substratum (Supplementary Information, Fig. S6A). Daughter cells were motile until they began to assemble their lorica. They did not swim but glided along the bottom of the well (Supplementary Information, Figs. S6B and S13) with maximal velocities of 60–70 μm min^−1^ (Supplementary Information, Fig. S9). Daughter cells therefore remained within the optical focus range of the oCelloScope. Some daughter cells did, however, move out of the filmed area before they settled, while others came into and settled within the filmed area. The elongated cytoplasmic strand did not always break and the daughter cell then settled in close proximity of its parental lorica (Supplementary Information, Fig. S9 and Supplementary Video S1). We also observed that *D. grandis* occasionally abandoned their lorica without going through a cell division (Supplementary Information, Fig. S11 and Supplementary Video S2). Protoplasts then became temporarily motile until they again settled and formed new lorica and resumed daughter cell formation. The resolution of the oCelloScope was insufficient to quantify the bacterial cells in the wells, and it was not clear to which extent also bacteria settled on the bottom of the wells.

### Single cell growth kinetics

**Figure 4:**
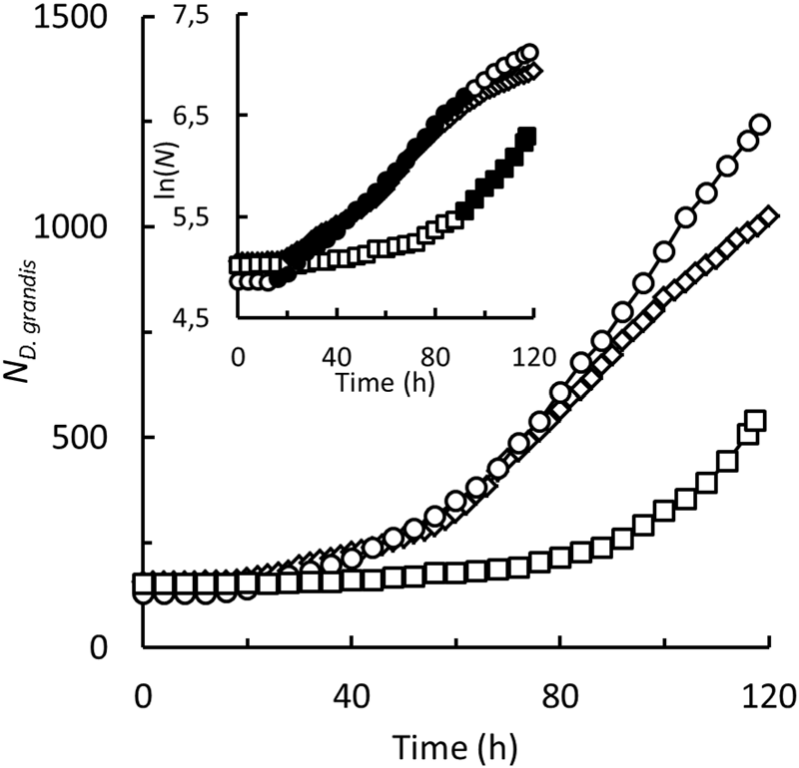
*Diaphanoeca grandis*. Number of individuals in filmed area in oCelloScope, grown on 3,000 (◇), 6,000 (○) and 12,000 (□) *Pantoea* sp. μL^−1^. Inset. ln(*D. grandis* numbers) vs. time, exponential growth phase indicated by solid symbols, slope of regression line equals specific growth rates, *µ* (Supplementary Information, Table S1).

Three *Diaphanoeca grandis* cultures were followed in the oCelloScope for a period of 6 days (Fig. 4), generating 1.9 Terabytes of image-data. Between 129 and 157 individual *D. grandis* settled on the bottom of the wells within the filmed area. After 16–20 h, the number of *D. grandis* started to increase in the cultures grown on initial *Pantoea* sp. concentrations of 3,000 and 6,000 μL^−1^. In the third culture where the initial *Pantoea* sp. concentration was 12,000 μL^−1^, the lag phase lasted approximately 92 h. The number of *D. grandis* increased 3.6–9.6 times, corresponding to 1.8–3.3 cell divisions (Supplementary Information, Table S1). Specific growth rates during exponential growth phases reached values of 0.49–0.66 day^−1^.

**Figure 5:**
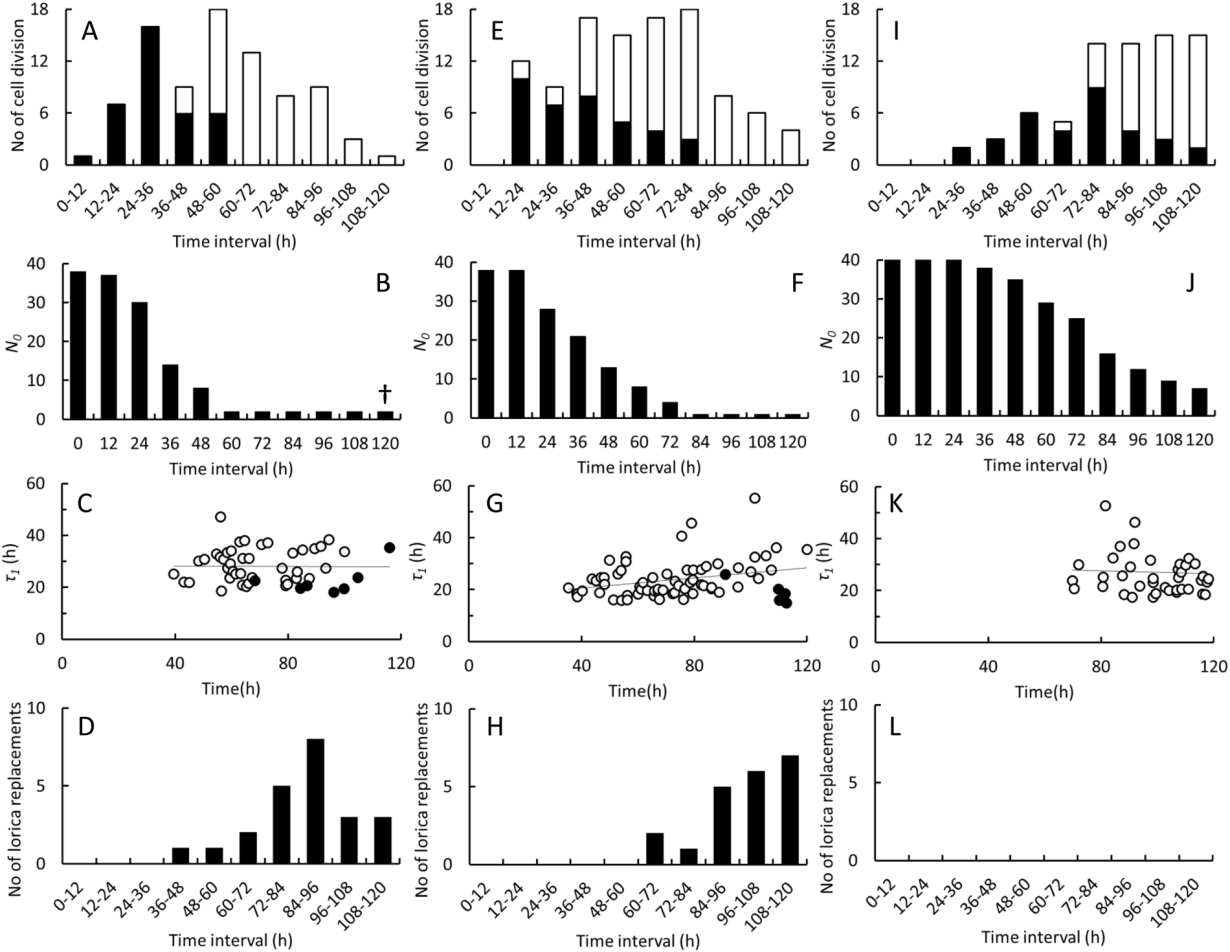
*Diaphanoeca grandis*. Data on cohorts of individuals inoculated and filmed in the oCelloScope and grown on 3,000 (38 individuals, **A**–**D**), 6,000 (40 individuals, **E**–**H**) and 12,000 (40 individuals, **I**–**L**) *Pantoea* sp. μL^−1^. **A**, **E** and **I**. Number of cell divisions within 12 h time intervals. Solid bars indicate first release of a daughter cell, open bars indicate release of subsequent daughter cells. **B**, **F** and **J**. Number of inoculated *D. grandis* that have not yet carried out a cell division. **C**, **G** and **K**. Time span between cell divisions, excluding the first cell division (○), *τ*_*1*_ and time span between replacement of a lorica and the following cell division (●). **D**, **H** and **L**. Number of protoplasts leaving and replacing their lorica within 12 h time intervals.

Figure 5 shows a number of observations made on cohorts of 38–40 parental *Diaphanoeca grandis* inoculated into the 3 cultures shown in Fig. 4. These individuals represented 24–31% of the total number of *D. grandis* that originally had settled within the filmed area. All individuals in the 3 cohorts were followed for 6 days and all individual life history events shown in Fig. 2 were recorded. There was considerable variation within each culture with respect to the point in time when each parental *D. grandis* produced its first daughter cell. The lower the initial *Pantoea* sp. concentration was, the earlier did daughter cell formation begin (Fig. 5A,E,I). Between 1 and 5 parental *D. grandis* did not produce daughter cells within the 6 days of the experiment (Fig. 5B,F,J). Most of these individuals maintained the same physical appearance as the active ones. Only 2 individuals in the culture grown at the lowest of the *Pantoea* sp. concentrations (Fig. 5B) lost their normal appearance and presumably died.

Most of the parental *D. grandis* in the cohorts produced several daughter cells (Fig. 5A,E,I), so each individual ended up producing 1–5 daughter cells in total (Supplementary Information, Fig. S10). On average, 2.1–2.7 daughter cells were produced by each of the parental *D. grandis*. The cultures inoculated into 3,000 and 6,000 *Pantoea* sp. μL^−1^ grew beyond the exponential growth phase (Fig. 4). In these 2 cultures, the rate of daughter cell formation reached a maximum of 15–18 daughter cells every 12 h for periods of time lasting 0.5 and 2 days, respectively. The time span between individual cell divisions, *τ*_1_ showed considerable variation (16–69 h) while the average value remained at the same level (26 ± 8 h) until the end of the experiment (Fig. 5C,G,K, and Table 1). In the cultures inoculated into 3,000 or 6,000 *Pantoea* sp. μL^−1^, it was therefore a decrease in the fraction of actively dividing individuals that resulted in the apparent decrease of specific growth rate after 80–90 h, rather than a decrease in the activity of all individuals in the cultures.

**Table 1:** *Diaphanoeca grandis*. Characteristic life times (average values ± standard deviation) of individual cell stages from 3 cultures grown in the oCelloScope on *Pantoea* sp. (Figs 5 and 6). Time span between cell divisions, *τ*_1_. Life time of stage with two cells (mother and daughter) inside the parental lorica, *τ*_2_. Life time of motile, non-loricate cell stage, *τ*_3_. Time expenditure for assembly of new lorica, *τ*_4_. Time span since last cell division when a protoplast abandons its lorica, *τ*_5_. Number of events, *n*.

	Events following cell divisions		Events following lorica abandoning	
	Characteristic life time	*n*	Characteristic life time	*n*
	h		h	
τ_1_	26 ± 8	157	21 ± 5	12
τ_2_	0.29 ± 0.10	261	—	—
τ_3_	0.64 ± 0.32	175	0.65 ± 0.37	37
τ_4_	0.29 ± 0.09	173	0.32 ± 0.13	31
τ_5_	59 ± 19	45	—	—

We also observed parental protoplasts abandoning their lorica (Fig. 5D,H,L and Supplementary Information, Fig. S11). These events began earlier in the culture inoculated into 3,000 *Pantoea* sp. μL^−1^ than in the culture inoculate into 6,000 *Pantoea* sp. μL^−1^ while none of the protoplasts abandoned their lorica in the culture inoculated into 12,000 *Pantoea* sp. μL^−1^. Before a protoplast abandoned its lorica, it had not produced a daughter cell for a period, *τ*_5_ of 59 ± 19 h (Table 1). After they had constructed a new lorica, daughter cell formation resumed after 21 ± 5 h, *n* = 12, a period of time quit similar to the characteristic time span between cell divisions, *τ*_1_ (Fig. 5C,G). When a protoplast abandoned its lorica, the formation of daughter cells was thereby delayed by more than 2 days.

**Figure 6:**
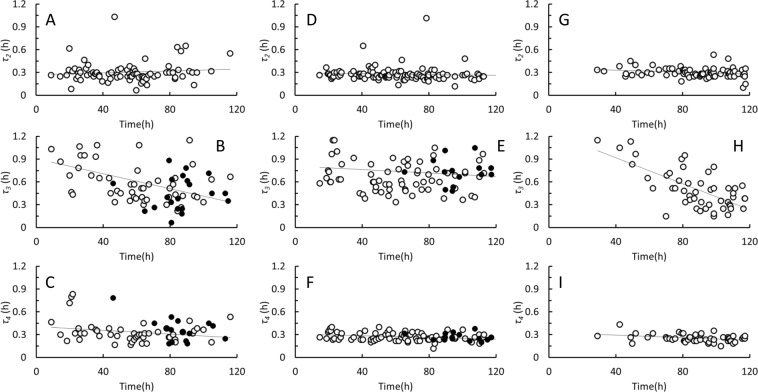
*Diaphanoeca grandis*. Data on cohorts of individual *D. grandis* inoculated and filmed in the oCelloScope and grown on 3,000 (38 individuals, **A**–**C**), 6,000 (40 individuals, **D**–**F**) and 12,000 (40 individuals, **G**–**I**) *Pantoea* sp. μL^−1^. **A**, **D** and **G**. Life time of stage with two cells (mother and daughter) inside the parental lorica, *τ*_2_. **B**, **E** and **H**. Life time of motile, non-loricate cell stage, *τ*_3_. **C**, **F** and **I**. Time expenditure for assembly of new lorica, *τ*_4_. Open symbols indicate events following cell divisions. Closed symbols indicate events that occurred after a protoplast had abandoned its lorica.

Figure 6 shows characteristic life times of the 3 distinguishable cell stages that follow a cell division in *D. grandis* (Fig. 2). The stage where two protoplasts (mother and daughter) reside inside the lorica after a cell division, *τ*_2_, lasted 0.29 ± 0.10 h (Table 1). The motile, non-loricate cell stage, *τ*_3_, lasted 0.64 ± 0.32 h, and finally the daughter cells used 0.29 ± 0.09 h to assemble their new lorica, *τ*_4_. All in all, daughter cells spend about 1.2 h to transform into mature, loricate choanoflagellates. The characteristic life times, *τ*_2_, and *τ*_4_, showed only little variation. Only on a few occasions did a daughter cell stay inside the parental lorica either considerably longer or shorter than the average value. Larger variations were observed in the lifetime of the motile, non-loricate daughter cells, *τ*_3_, and there may have been a weak tendency for motile, non-loricate daughter cells to exist for shorter periods of time during the late phases of the cultures.

### Linking growth kinetics at the culture and at the single cell level

**Figure 7:**
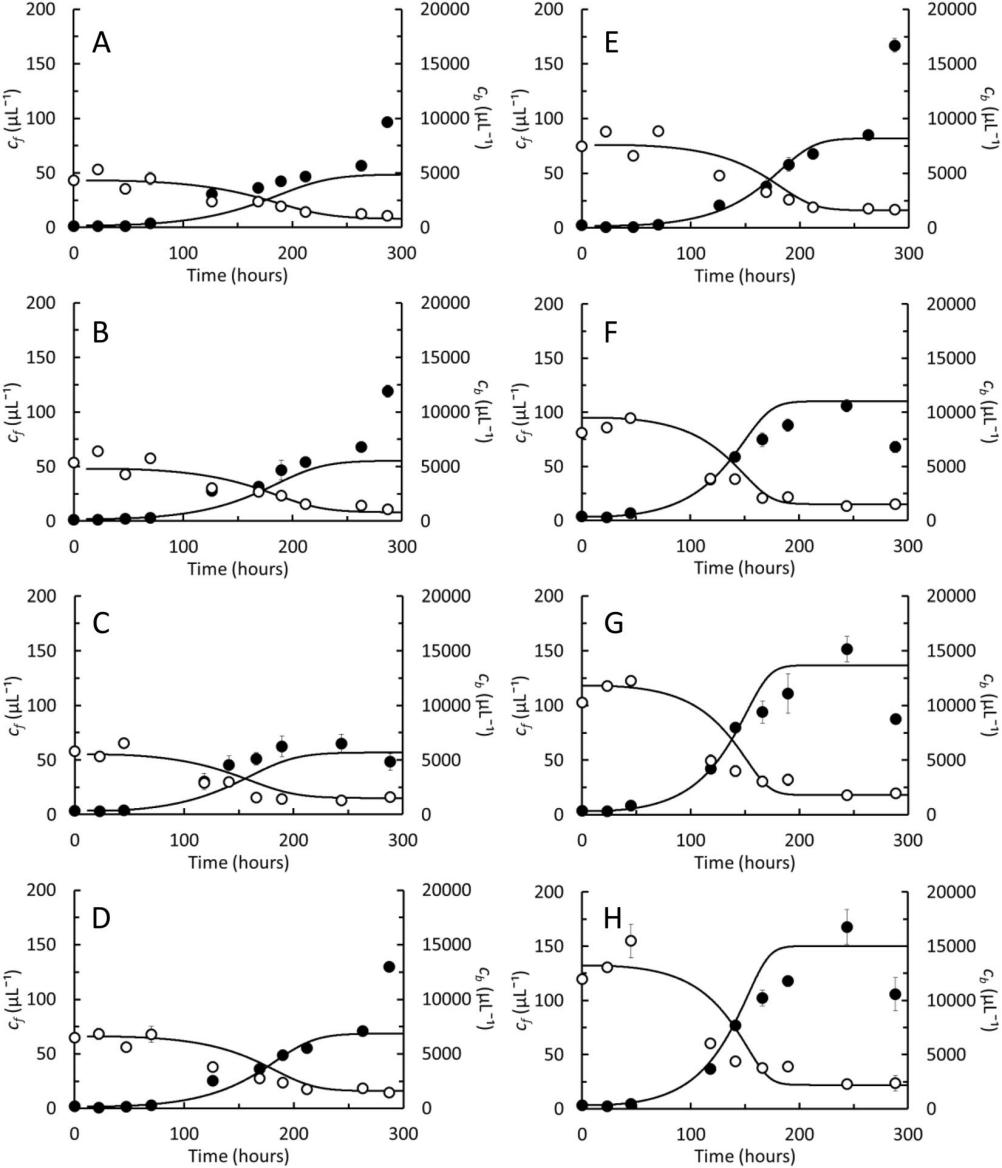
*Diaphanoeca grandis*. Batch cultures grown on *Pantoea* sp. at initial concentrations of 4,500 (**A**), 5,000 (**B**), 5,500 (**C**), 7,000 (**D**), 9,000 (**E**), 9,500 (**F**), 12,000 (**G**) and 13,000 (**H**) *Pantoea* sp. μL^-1^. Concentrations of *D. grandis*, *c*_*f*_ (●) and bacterial prey *Pantoea* sp., *c*_*b*_ (○). All data points are average measurements from 3 replicate cultures ± standard error. Curves show the concentrations of *D. grandis* and bacteria modelled by Eqs. 2–7, using the characteristic life time of the different cell stages listed in Table 1.

In order to test if the observations made on individual *D. grandis* observed in the oCelloScope would be able to explain the progression of cultures grown at larger scale, we carried out a series of 50 mL batch cultures where *D. grandis* was grown on 8 different initial *Pantoea* sp. concentrations between 4,500 and 12,000 μL^−1^. At each *Pantoea* sp. concentration, 3 replicate cultures were carried out. The *Pantoea* sp. concentrations were selected within the range where the specific growth rate was maximal (Fig. 3D). Concentrations of *D. grandis* and *Pantoea* sp. were quantified daily and compared to concentrations predicted by Eqs. 2–7 (cell numbers were divided by culture volume to predict cell concentrations), while the rate constants, *k*_0_ - *k*_4_ were based upon single cell kinetics (Table 1). In all cultures, the predicted concentrations of *D. grandis* as well as *Pantoea* sp. were well in line with experimentally determined cell concentrations (Fig. 7). Predicted concentrations of the individual cell stages can be seen in Supplementary Information, Fig. S1. The mature, loricate cell stage was always dominating because the other cell stages were comparatively short lived (Table 1).

## Discussion

*Diaphanoeca grandis* grew in batch cultures on *Pantoea* sp. as its sole prey. The immobility of loricate *D. grandis* and the close association to the bottom substratum of all cell stages made *D. grandis* an ideal candidate to be examined in the oCelloScope. Growth kinetics observed in 200 μL cultures analysed at the individual cell level in the oCelloScope (Figs 4–6) was in agreement with kinetics observed in 50 mL cultures analysed at the culture level by flow cytometry (Fig. 7), indicating that *D. grandis* behaved similarly in both culture types. These analyses provided novel insights into the life history of *D. grandis* with relevance to our understanding of the role of *D. grandis* in the environment. The analyses also demonstrated an unforeseen high degree of heterogeneity within the cultures.

*D. grandis* is a common member of marine planktonic communities^1,2,4^. Loricate *D. grandis* have been described as slow swimmers^3^ but can also be found attached to detritus^31^. The isolate used in this study was isolated from the water column, albeit sampled at shallow waters at a coastal location. All observations in the laboratory seemed, however, to contradict that *D. grandis* should have a planktonic life style. All attempts to grow *D. grandis* in stirred suspension cultures failed, irrespectively of stirring method and long term observations on individual cells showed that loricate *D. grandis* are immobile and reside on the bottom substratum. Individuals remained at the same position for the experimental period of 6 days, except when protoplasts abandoned their lorica and temporarily became motile. Cells were, in most cases, motile for less than one hour (Fig. 6B,E,H). Since the elongated cytoplasmic strand, used to push out daughter cells from the parental lorica^14^, did not always break and daughter cells then settled in proximity of their parental lorica (Supplementary Information, Fig. S9), individual *D. grandis* did not distribute themselves homogeneously at the bottom surface of the 96-well plates, in where they were grown. Some tended to form smaller groups of up to 10–20 individuals (Supplementary Information, Figs S4-S5). Also when the elongated cytoplasmic strand did break, and the daughter cell became fully motile, they did not swim but glided on the bottom substratum. Sediments and other surfaces such as e.g. marine snow may therefore be important and somewhat overlooked habitats for *D. grandis*.

*D. grandis* was not attached to the bottom substratum and its lorica is also without a stalk, which secures other *Diaphanoeca* species a fixed orientation perpendicularly to the substratum^22^. Occasionally we observed that motile, non-loricate daughter cells collided with mature lorica, which were then either pushed or rolled slightly out of the way (Supplementary Information, Fig. S13). It may therefore be that *D. grandis* is easily brought temporarily into suspension in turbulent waters where it can then be found in the water column, often near the coast^1,2,4^. Several fluid flow models have been used to predict magnitude and direction of fluid flows generated by *D. grandis* and other choanoflagellates that are either freely suspended in the water column^13,32^ or anteriorly attached to a solid substratum with their longitudinal axis oriented perpendicularly to the surface^32,33^. Most *D. grandis* settled with their longitudinal axis in parallel to the bottom substratum (Supplementary Information, Fig. S6), and therefore in a different orientation than normally expected for a choanoflagellate.

We were able to grow *D. grandis* successfully only in non-stirred cultures, mixed only before samples were collected once or twice per day. In between, *D. grandis* concentrated on the bottom of the culture flasks. Inhomogeneous distribution of cells may therefore have influenced kinetic and stoichiometric characteristics. Ingestion rate (53 *Pantoea* sp. day^−1^) and specific growth rate (0.72 day^−1^) were low compared to the maximal rates (40 *Pseudomonas* h^−1^ and 0.12 h^−1^ (2.88 day^−1^), respectively, observed before in *D. grandis*^3^. In *Stephanoeca diplocostata*, a second loricate choanoflagellate, the maximal specific growth rate was 1.9 day^−1^, but at bacterial concentrations of 100,000 per μL^15^, which is 5 times higher than the bacterial concentrations tolerated by *D. grandis* without negative effects on growth (Fig. 3D). The cell cycle also proceeds at a slightly faster rate in *S. diplocostata*, in which species the total time from a protoplast visibly starts to divide until the daughter cell is released has been measured to 12 min (Leadbeater 1976) as compared to 0,29 h (17 min) in *D. grandis* (Table 1). The average protoplast volume of 60 ± 28 μm^3^ was, however, close to earlier estimates in *D. grandis* (70–83 μm^3^)^3,13^ but covered at wide span from 15–160 μm^3^ (Supplementary Information Fig. S3). The average protoplast size of *D. grandis* decreased considerably during batch cultivation. *D. grandis* may therefore need more than 74 *Pantoea* sp. to produce one daughter cell (Fig. 3B) during steady state situations where the cell size is preserved.

Analyses on individual cells furthermore revealed that balanced growth in batch cultures of *D. grandis* was not necessarily obtained although specific growth rates and growth yields measured at the culture level reached constant values. Balanced growth is, however, a common presumption in models used for determination of specific rates of e.g. growth and food uptake in growing microbial cultures^25,34^. *D. grandis* could be grown only at a narrow range of bacterial prey concentrations that either did not limit or inhibit growth (Fig. 3D). Cultures inoculated at 2–10 *D. grandis* μL^−1^ and 5,000–12,000 *Pantoea* sp. μL^−1^ completed 3–6 generations. Cell counts indicated that growth was exponential (Fig. 3B), and yields became constant after a lag phase (Fig. 3C). Still, analyses of individual cells displayed substantial heterogeneity. Figure 4 indicates that *D. grandis* cultures grown at initial *Pantoea* sp. concentrations of 3,000 or 6,000 μL^−1^ entered the exponential growth phase in less than 24 h. Some of the parental *D. grandis* did, however, not produce their first daughter cell until after 48–72 h (Fig. 5B,F). Also the time span between consecutive releases of daughter cells, *τ*_1_ was variable, ranging from 16–69 h (Fig. 5C,G,K). There were therefore parental *D. grandis* that produced 2 or 3 daughter cells, and there were daughter cells that matured and began to divide, before the last of the parental *D. grandis* produced their first daughter cell. This led to a large degree of heterogeneity also in the productivity among the parental *D. grandis* (Supplementary Information, Fig. S10).

Although the time span between releases of daughter cells showed considerable variability, *τ*_1_ seemed not to be a function of time within the 6 days the cultures were monitored (Fig. 5C,G,K). The decrease in the rate of daughter cell formation, which occurred after 60–84 h at 3,000 and 6,000 *Pantoea* sp. μL^−1^ (Fig. 5A,E), and the subsequent decrease in specific growth rate (Fig. 3, inset) was rather a consequence of protoplasts abandoning and replacing their lorica (Fig. 5D,H). Before protoplasts replaced their lorica, they experienced a more than 2-day break in the formation of new daughter cells. Protoplasts began to replace their lorica earlier when grown on 3,000 *Pantoea* sp. μL^−1^ than on 6,000 *Pantoea* sp. μL^−1^ and more than half of the *D. grandis* initially inoculated into the cultures ended up replacing their lorica in these two cultures. This behaviour was not observed in the culture grown on 12,000 *Pantoea* sp. μL^−1^, which did not grow beyond the exponential growth phase before it was terminated. Protoplasts did not differ in appearance or motion pattern (Supplementary Information, Figs. S11 and S12) from the motile, non-loricate daughter cells. It could be observed that they are carrying costal strips (Supplementary Information, Fig. S11) as also daughter cells do^11,14^. Naked *Stephanoeca diplocostata*, deprived of their lorica, is able to resynthesize a new lorica^35^ and this could potentially have been a possibility also in *D. grandis*. The abandoning protoplasts resembled the daughter cells also with respect to size, shape, and motion pattern (Supplementary Information, Figs. S7-S9 and S11-S12). Some protoplasts stayed active within the same lorica during the entire experimental period, showing that the lifetime of a functional lorica exceeds 6 days, although silica may continuously dissolve from the costal strips, limiting the lifetime of a lorica^36^.

Figure 5 shows that lorica replacements can be frequent events in *D. grandis* and may be associated to prey availability. Protoplasts did, however, most likely start to replace their lorica before *Pantoea* sp. was depleted from the cultures since they managed to resume daughter cell formation after they had reconstructed a new lorica (Fig. 5C,G). What actually triggered protoplasts to abandon and replace their lorica remain unresolved, why this phenomenon was not incorporated in the growth model (Eqs. 2–7). Monod kinetics (Supplementary Information, Eq. S1) describes the transient decrease in specific growth rate, which takes place when batch cultures progress from exponential to stationary phase due to food limitation and the time span between cell divisions is prolonged for all cells in a culture. In *D. grandis* cultures, this interpretation is too simplistic since the specific growth rate is also affected when protoplasts abandon and replace their lorica. It has, to our knowledge, not previously been described that *D. grandis* can replace its lorica but this may provide *D. grandis* an opportunity to temporarily regain motility and move to new and potentially better positions.

More than 115 species of loricate choanoflagellates have been identified based on morphology, and from more than 20 species are also sequence data available^37^. Growth, feeding, and life history have been described in only a few of these. In this study, we have linked single cell growth kinetics to growth kinetics at the culture level in *D. grandis*. The structured growth model (Eqs. 2–7) provides a more detailed and comprehensive interpretation of growth kinetics in *D. grandis* than the Monod model^3^. The characteristic time span between cell divisions, *τ*_1_ was much longer than the characteristic life times, *τ*_2_ - *τ*_4_ associated with the different daughter cell stages (Table 1), meaning that the maximum specific growth rate of *D. grandis* is mainly controlled by the frequency by which mature individuals form new daughter cells, *k*_1_. When protoplasts abandoned and replaced their lorica, daughter cell formation slowed down considerably, and the event represents a substantial investment in time, indicating that the ability to regain motility and obtain the opportunity to move to a new location is of importance to *D. grandis*. The combination of analyses at the culture level and at the individual cell level furthermore demonstrated an extensive degree of heterogeneity in *D. grandis* cultures, even during exponential growth phases. Individual cell performances may therefore not necessarily be well represented by kinetic data representing the average performances of all cells in cultures of *D. grandis* and possibly also other microorganisms.

## Supplementary information

Supplementary Information

Supplementary Video S1

Supplementary Video S2

## Data Availability

The datasets generated during and/or analysed during the current study are available from the corresponding author on reasonable request.
