# Supplementary material for: Growth and single cell kinetics of the loricate choanoflagellate Diaphanoeca grandis
Source: Sci Rep. 2019 Oct 10;9:14543. doi: 10.1038/s41598-019-50998-0 (PMC6787020; doi:10.1038/s41598-019-50998-0)
Supplement: Supplementary file 1 — Supplementary Information [file 41598_2019_50998_MOESM1_ESM.pdf]

Supplementary Information

Growth and single cell kinetics of the loricate choanoflagellate *Diaphanoeca grandis*

Niels Thomas Eriksen[1,*], Jakob Tophøj[1], Rasmus Dam Wollenberg[1], Teis Esben Sondergaard[1], Peter Funch[2], Per Andersen[2]
[1]Department of Chemistry and Bioscience, Aalborg University, Fredrik Bajers Vej 7H, DK-9220 Aalborg, Denmark,
[2]Department of Bioscience, Aarhus University, Ny Munkegade 116, DK-8000 Aarhus, Denmark

Kinetic growth model. The specific growth rate*,* μ of *D. grandis* has previously been described by the Monod type kinetics with suspended bacteria as the growth limiting food source at concentrations c_b

$$\mu = \mu_{max}\left(\frac{c_b}{K_b + c_b}\right) \tag{S1}$$

where μ_{max} is the maximum specific growth rate and K_b is the half-saturation constant.

Inhibitory effects from high bacterial concentrations are not well understood but may e.g. be described by a sigmoidal dose-response curve

$$\mu = \mu_{max}\left(1 - \frac{1}{1 + e^{(EC_{50} - c_b)\cdot H}}\right) \tag{S2}$$

where EC_{50} is the effective bacterial concentration at which the specific growth rate is reduced to half its maximal value, and the coefficient H defines the steepness of the slope of the curve.

Eqs. S1 and S2 predict that maximum specific growth rates will be obtained at indefinite or zero bacterial concentrations, respectively. A combination of the two equations will result in a kinetic model that describe the limiting as well as the inhibitory effects of bacterial concentration on the specific growth rate

$$\mu = \mu_{max}\left(\frac{c_b}{K_b + c_b}\right)\left(1 - \frac{1}{1 + e^{(EC_{50} - c_b)\cdot H}}\right) \tag{S3}$$

In Eq. S3 (identical to Eq. 1) should μ_{max} be considered a theoretical parameter that in reality may be unattainable by *D. grandis* cultures.

Numerical solution to growth model. Numerical solutions to the structured growth model, Eqs. 2-7 are shown in Eqs. S4-S9. Cell numbers, *N* have been converted to cell concentrations, *c* by division with the liquid volume of the culture. Then, the numerical solutions to Eqs. 2-7 are

$$c_{0,t+\Delta t} = c_{0,t} - k_0 \cdot c_{0,t} \cdot \Delta t \tag{S4}$$

$$c_{m,t+\Delta t} = c_{m,t} + \left(k_0 \cdot c_{0,t} + k_4 \cdot c_{l,t}\right) \cdot \Delta t \tag{S5}$$

$$c_{d,t+\Delta t} = c_{d,t} + \left(k_0 \cdot c_{0,t} + k_1 \frac{c_{b,t}}{K_b + c_{b,t}} c_{m,t} - k_2 \cdot c_{d,t}\right) \cdot \Delta t \tag{S6}$$

$$c_{n,t+\Delta t} = c_{n,t} + \left(k_2 \cdot c_{d,t} - k_3 \cdot c_{n,t}\right) \cdot \Delta t \tag{S7}$$

$$c_{l,t+\Delta t} = c_{l,t} + \left(k_3 \cdot c_{n,t} - k_4 \cdot c_{l,t}\right) \cdot \Delta t \tag{S8}$$

$$c_{b,t+\Delta t} = c_{b,t} - Y_{f/b}^{-1}\left(c_{f,t+\Delta t} - c_{f,t}\right) \tag{S9}$$

Eqs. S4-S9 were solved sequentially in time intervals Δt = 0.2 h, in order to construct the growth curves in Figs. 7 and S1.

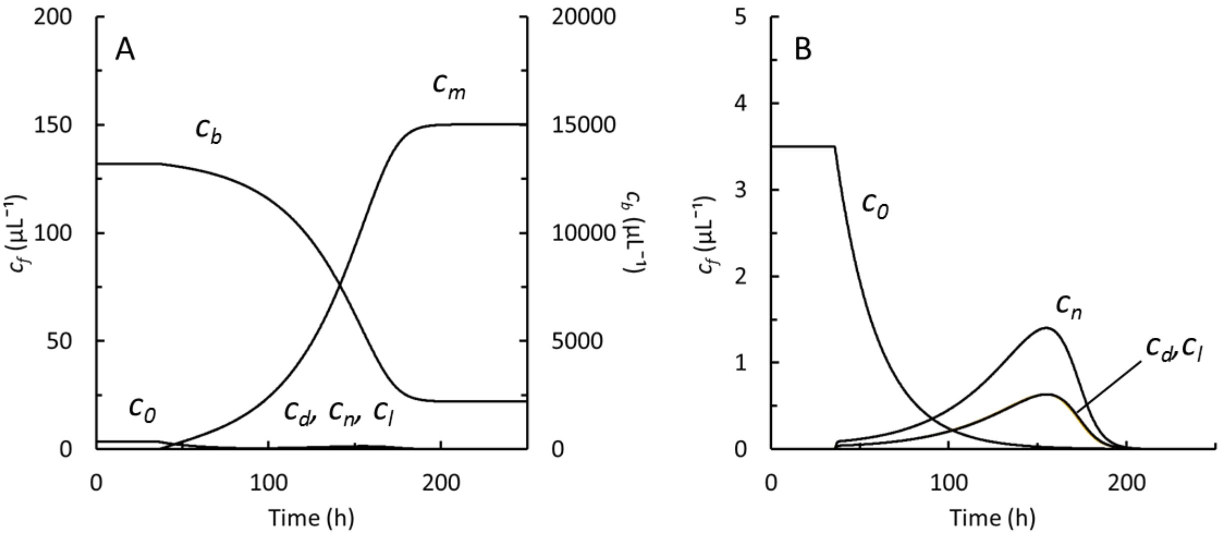

Figure S1. A. *Diaphanoeca grandis*. Concentrations of different flagellate (*D. grandis*) cell stages and bacterial prey (*Pantoea* sp.) simulated by Eqs. S4-S9. The simulation corresponds to the one in Fig. 7H. Inoculated cells that have not yet divided, c_0. Mature, loricate cells, c_m. Dividing cell stage with 2 protoplasts inside the lorica, c_d. Motile, non-loricate cell stage, c_n. *Pantoea* sp., c_b. Lorica forming cells, c_l. B. Same simulation as in A but on an expanded scale (c_d and c_l cannot be discriminated as concentrations of these 2 cells stages are almost identical due to their similar characteristic life time, τ_2 and τ_4).

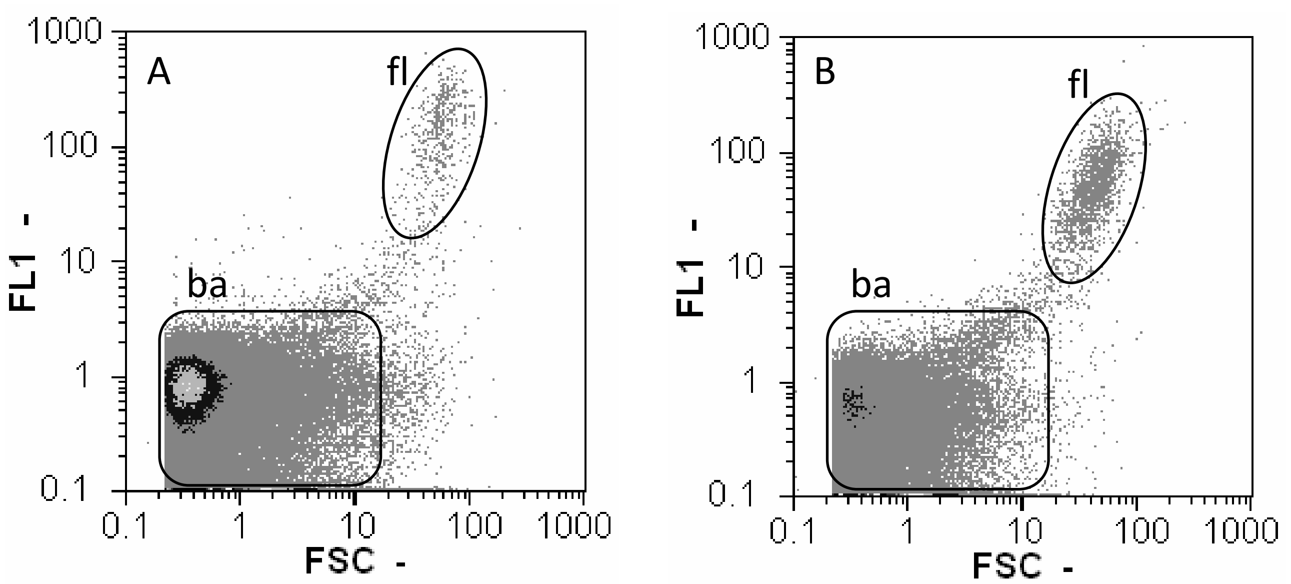

Figure S2. A. *Diaphanoeca grandis.* Flow cytometric analysis of green fluorence (FL1) vs. forward scatter (FSC) of bacterial cells, ba, and flagellate cells, fl, in culture grown on initial concentration of 14 000 *Pantoea* sp. µL^{-1}. A. Analysis made Day 3. B. Analysis made Day 5.

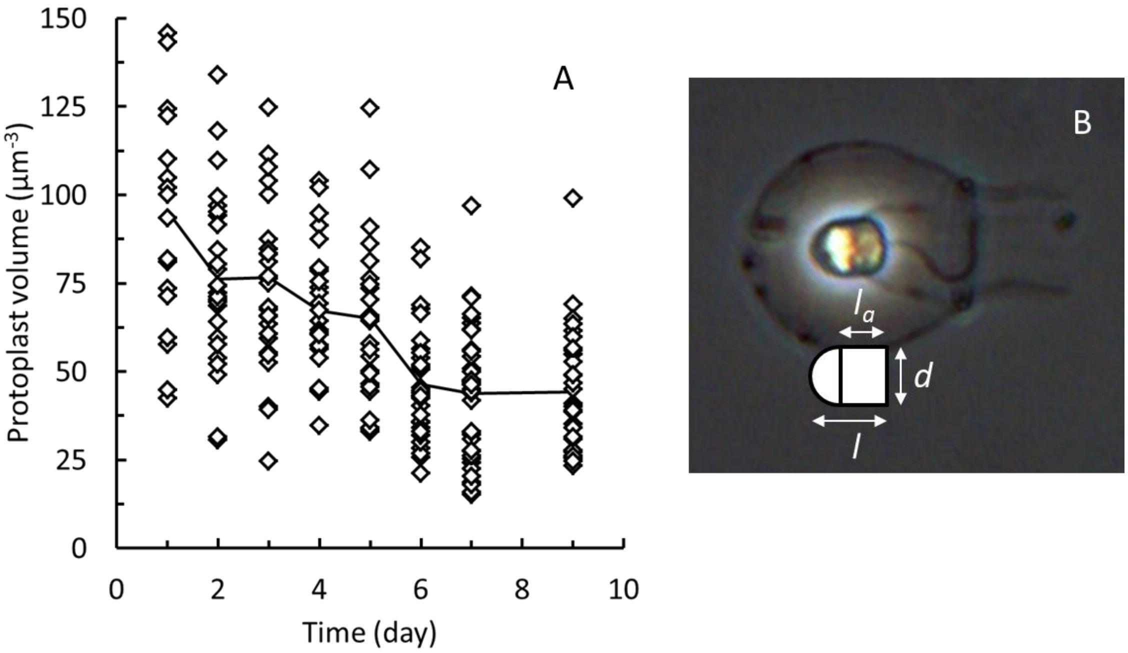

Figure S3. *Diaphanoeca grandis*. A. Protoplast volume estimated during batch cultivation. Solid line indicates average protoplast volume at Day 1-9. B. Protoplast dimensions i.e. total length, *l*, length of apical part, l_a, and diameter, *d*, were estimated in Fiji ImageJ ver. 2.0.0 from phase contrast micrographs of cells sampled from the culture. The volume of the apical part of the protoplast was estimated as cylinder, the volume of the frontal part as half a sphere, and the volume of the protoplast, *V*, was then calculated as

$$V = \pi \left(\frac{d}{2}\right)^2 l_a + \frac{1}{2} \cdot \frac{4}{3} \pi \left(\frac{d}{2}\right)^3 \tag{S10}$$

with symbols as indicated in B.

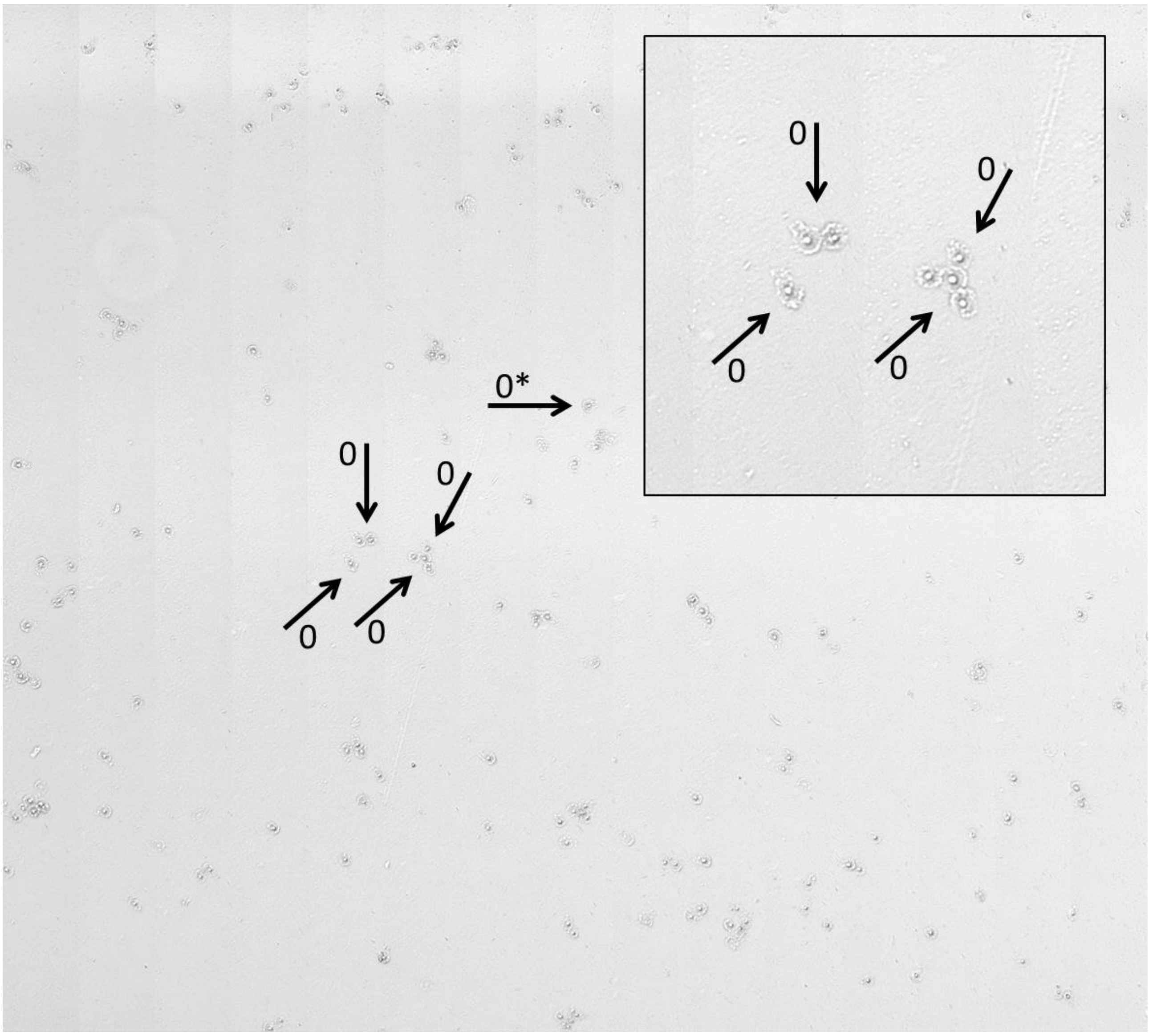

Figure S4. *Diaphanoeca grandis*. First image of culture grown at 3,000 *Pantoea* sp. μL^{-1} recorded in the oCelloScope (Fig. 4). The image covers an area of 1,300 µm × 1,200 µm. Arrows point at parental *D. grandis* inoculated into the culture marked 0 as they had not yet divided (cell stages described in Fig. 2). Inset shows same individual at an expanded scale. Individual marked 0* is described in further details in Figs. S7-S9 and S11-S12.

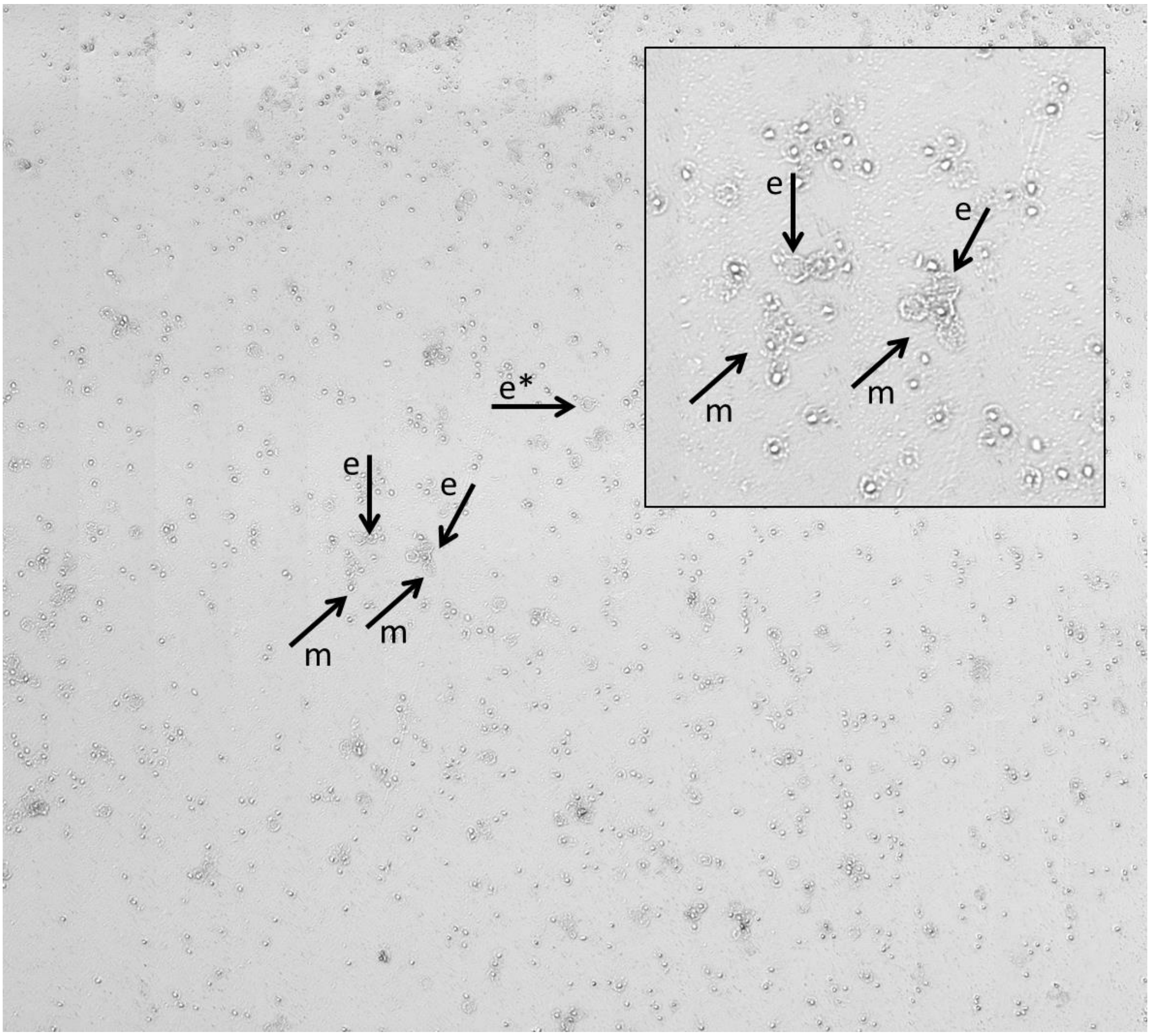

Figure S5. *Diaphanoeca grandis*. Last image of culture grown at 3,000 *Pantoea* sp. μL^{-1} recorded in the oCelloScope after 118.3 h (Fig. 4). The image covers an area of 1,300 µm × 1,200 µm. Arrows indicate the same 4 parental *D. grandis* as in Fig. S4. Inset shows same individuals at an expanded scale, e indicates empty lorica that have been abandoned by their protoplasts, m indicates mature, loricate *D. grandis* (cell stages described in Fig. 2). Individual marked e* (empty lorica) is described in further details in Figs. S7-S9 and S11-S12.

Table S1. *Diaphanoeca grandis*. Overall data from 3 cultures grown in the oCelloScope (Fig. 4). Initial concentrations of *Pantoea* sp., $c_{b,0}$. total number of *D. grandis* in the filmed area at t = 0, N_0 and at t = 120 h, N_{120}. Total increase in numbers of *D. grandis* and cell doublings during 120 h. Length of lag phase until exponential growth, and specific growth rate, μ during exponential growth phases.

$c_{b.0}$ µL^{-1}	N_0	N_{120}	Increase	Doublings	Lag phase h	μ day^{-1}
3,000	157	1025	6.5	2.7	20	0.49
6,000	129	1244	9.6	3.3	16	0.59
12,000	152	541	3.6	1.8	92	0.66

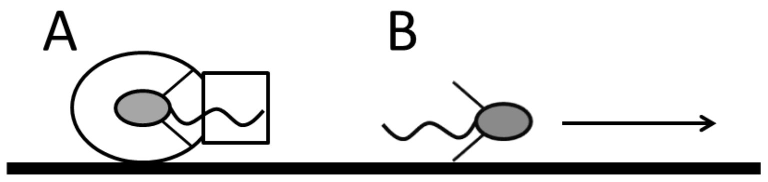

Figure S6. *Diaphanoeca grandis*. A. Common orientation of immobile, loricate *D. grandis* on bottom substratum. B. Motile, no-loricate cell gliding on the bottom substratum.

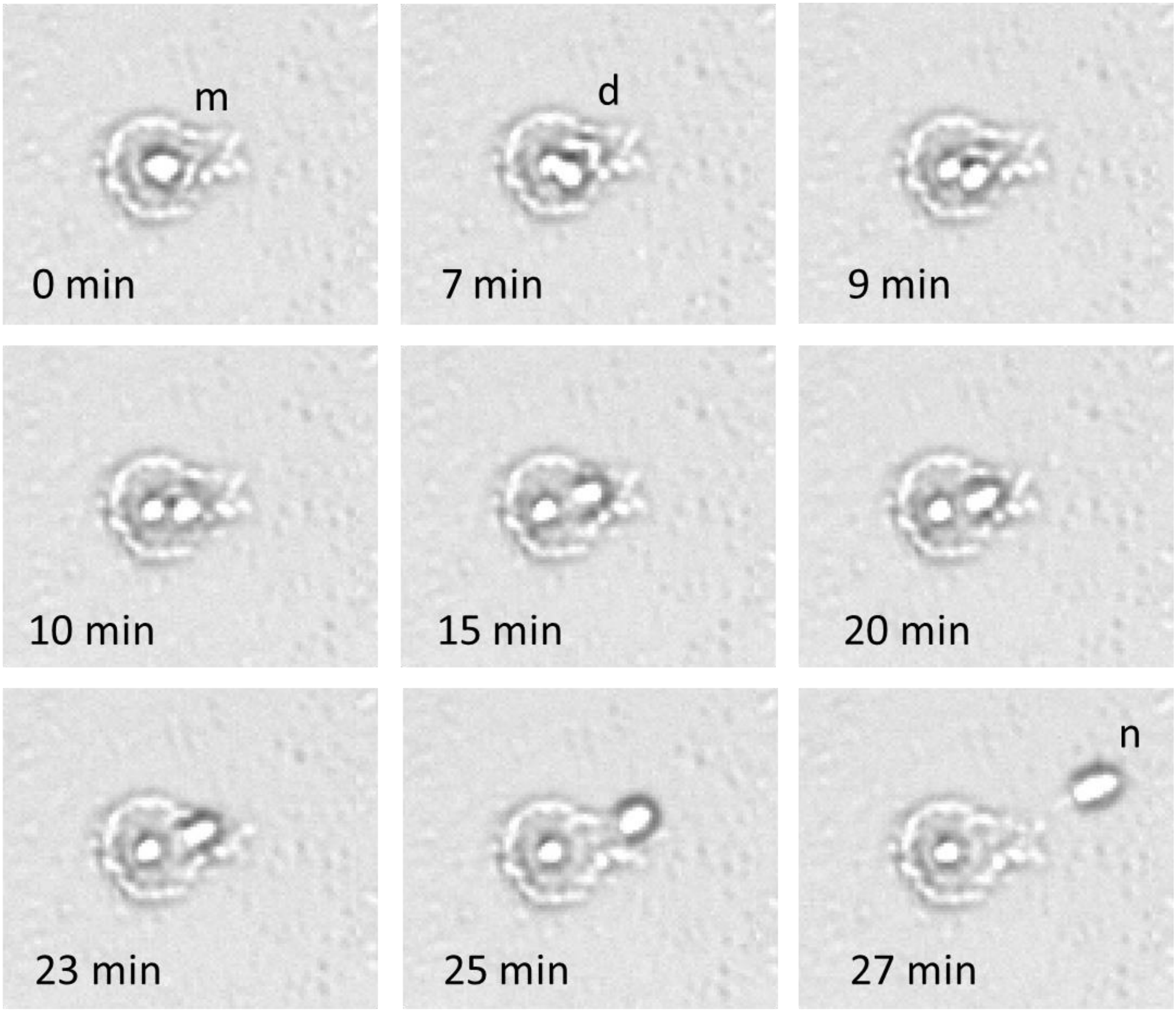

Figure S7. *Diaphanoeca grandis*. Time series of images of dividing protoplast 2.6 days after the culture had been inoculated into the oCelloScope. The first image of mature cell, m is obtained 7 min before cells were visibly dividing, d. After 27 min the non-loricate daughter protoplast, n, abandoned its parental lorica (cell stages described in Fig. 2).

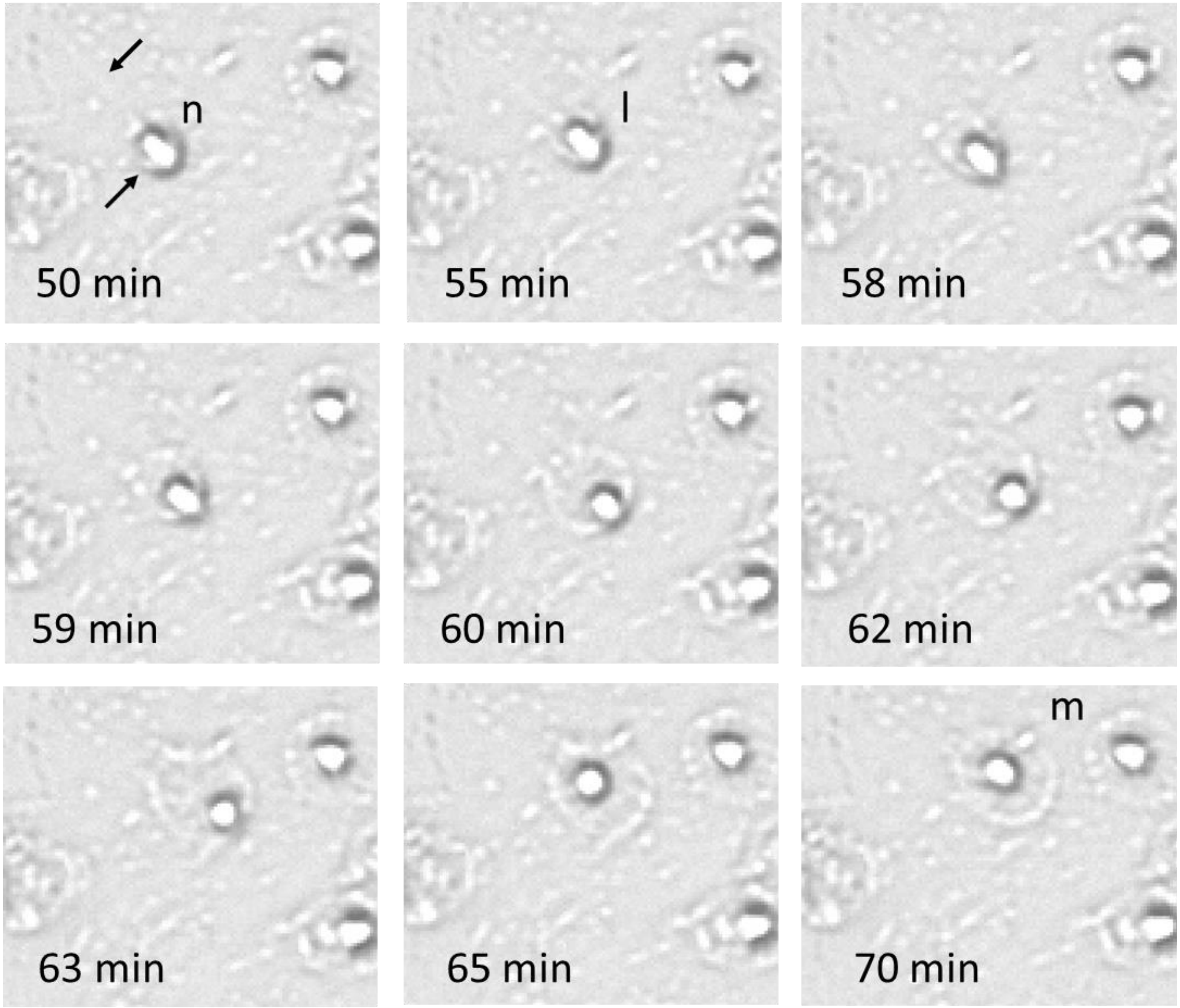

Figure S8. *Diaphanoeca grandis*. Time series of images of daughter cell forming a lorica 2.6 days after the culture had been inoculated into the oCelloScope. The individual is the daughter cell in Fig. S7. The first image of the daughter cell, n, is obtained 50 min after the first image in Fig. S7. The daughter cell began assembling its lorica after 55 min, which after 70 min was completed, and a mature *D. grandis*, m, was formed (cell stages described in Fig. 2). The protoplast localizes itself differently within the lorica when it is being assembled compared to after the lorica has been completed. Arrows in the image obtained at 50 min indicate the elongated cytoplasmic strand connecting mother and daughter cell, which in this event did not break, and what presumably are costal strips carried on the surface of the motile, non-loricate daughter cell. The daughter cell settled with a distance less than 50 µm from the parental lorica, although it travelled more than 3 times this distance (Fig. S9C). The sequence is also shown in Supplementary Video S1.

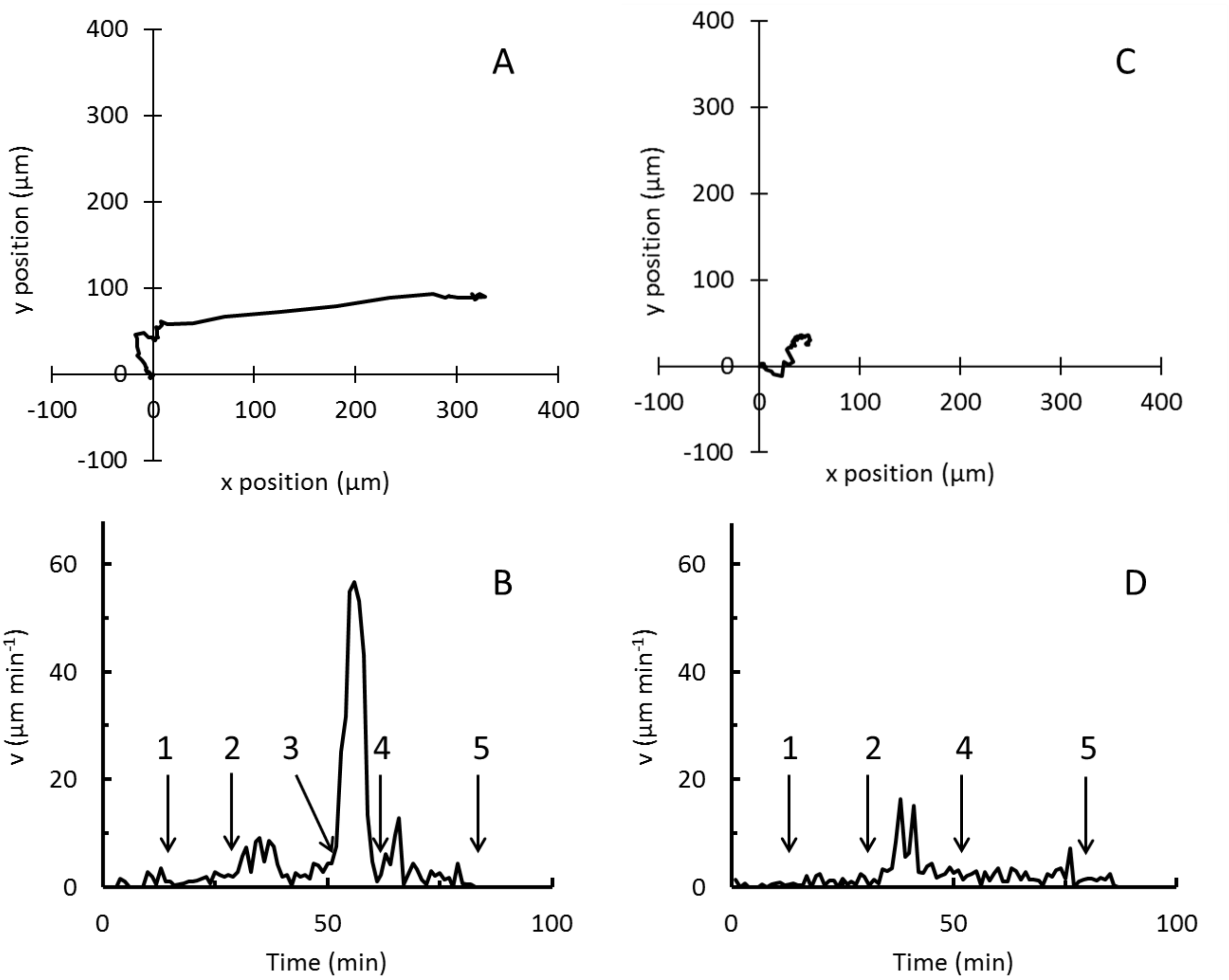

Figure S9. *Diaphanoeca grandis*. Examples of trajectories (A and C, parental lorica is positioned in (0, 0)) and velocities (B and D) of motile, non-loricate daughter cells, in one event where the elongated cytoplasmic strand came apart and the daughter cell was released (A and B, total distance travelled = 480 µm) and in one event where the elongated cytoplasmic strand did not break (C and D, same daughter cell as in Figs. S7 and S8, total distance travelled = 183 µm). The following events have been indicated by numbers: 1, two cells visible within parental lorica. 2, non-loricate daughter protoplast leaves parental lorica. 3, elongated cytoplasmic strand breaks. 4, daughter cell starts assembling its new lorica. 5, the new lorica is completed.

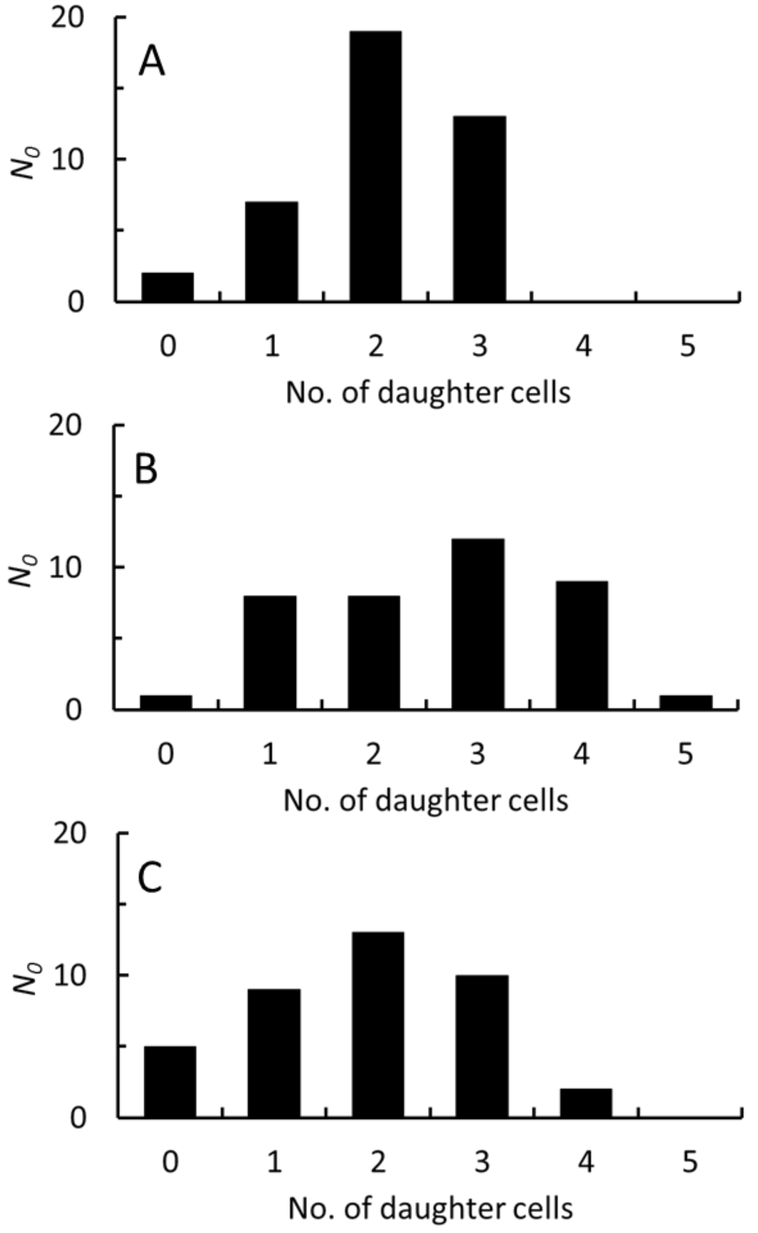

Figure S10. *Diaphanoeca grandis*, total number of daughter cells produced by individual *D. grandis* inoculated and filmed in the oCelloScope and grown on 3,000 (N_0 = 38, A), 6,000 (N_0 = 40, B) and 12,000 (N_0 = 40,C) *Pantoea* sp. µL^{-1} (Figs. 4-6).

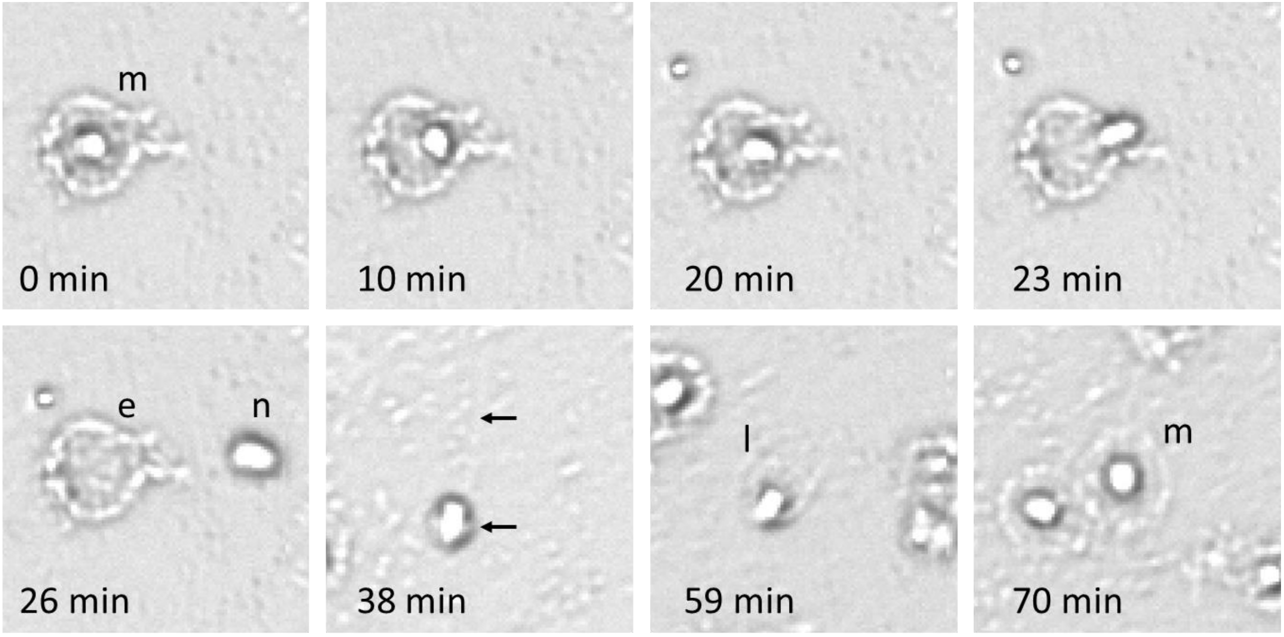

Figure S11. *Diaphanoeca grandis*. Time series of images of protoplast of mature *D. grandis*, m, abandoning its lorica 3.8 days after the culture had been inoculated into the oCelloScope. The individual is the same as in Fig. S7. The non-loricate protoplast, n, abandoned and left behind the empty lorica, e, 26 min into the sequence. It began to assemble a new lorica, l, 59 min into the sequence. After 70 min the new lorica was completed, and a mature *D. grandis*, m, was again formed (cell stages described in Fig. 2). The motile, non-loricate cell dragged behind itself an elongated cytoplasmic strand although there was no mother cell to connect to, and it carried what appeared to be costal strips, both structures indicated by arrows in the image taken 38 min into the sequence. The sequence is also shown in Supplementary Video S1.

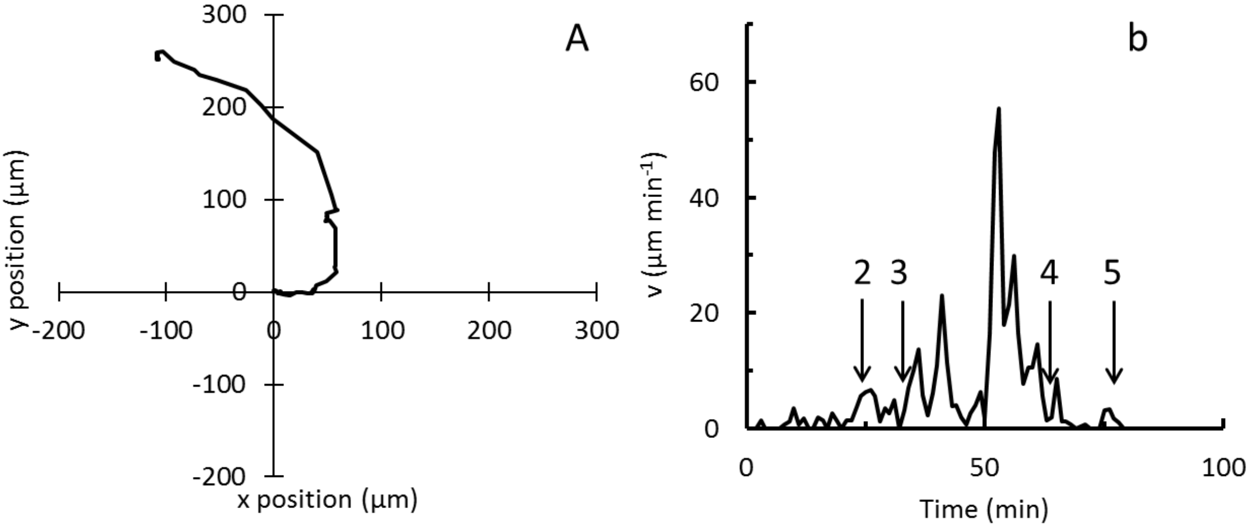

Figure S12. *Diaphanoeca grandis*. Trajectory (A, parental lorica positioned in (0, 0), total distance travelled = 456 µm) and velocity (B) of motile, non-loricate cell after it had abandoned its lorica. Same individual as in Fig. S11. The following events have been indicated by numbers: 2, protoplast leaves parental lorica. 3, elongated cytoplasmic strand breaks. 4, protoplast starts assembling its new lorica. 5, the new lorica is completed.

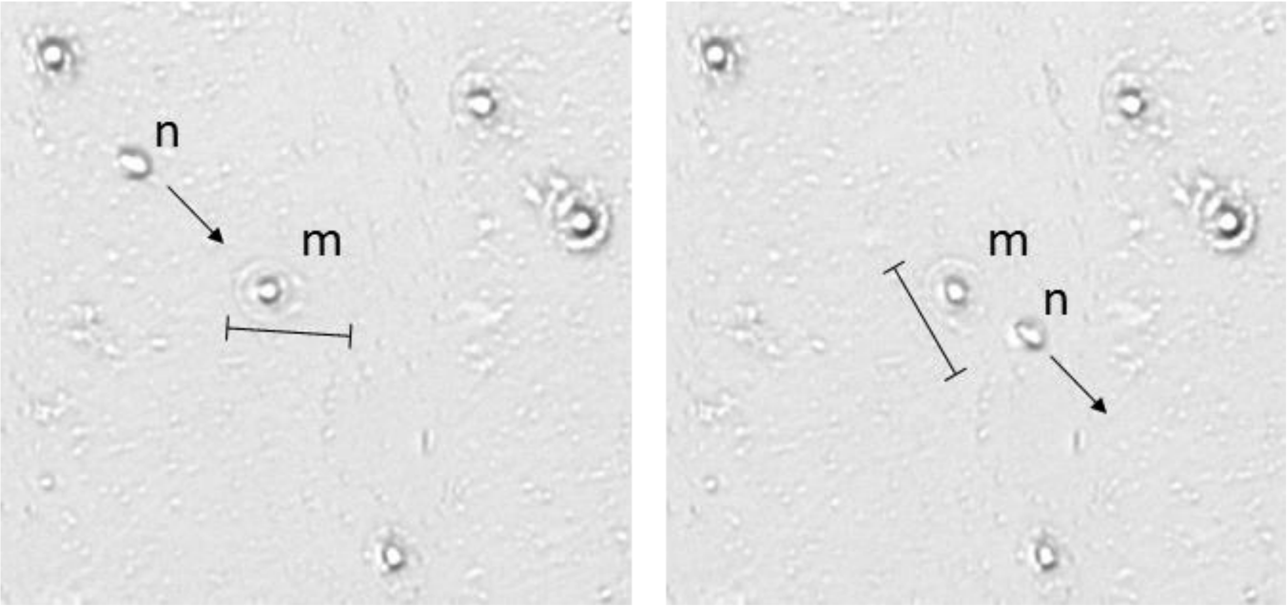

Figure S13. *Diaphanoeca grandis*. Images recorded at a 1 min interval showing motile, non-loricate daughter cell, n colliding with an immobile mature, loricate *D. grandis*, m. Arrow indicates the direction of movement by the daughter cell. Straight line indicates the orientation of the longitudinal axis of the mature *D. grandis*, which is pushed into a new orientation by the collision.

Supplementary Video S1. *Diaphanoeca grandis*. Dividing protoplast 2.6 days after the culture had been inoculated into the oCelloScope. Same parental cell indicated by arrow as in Figs. S7, and same daughter cell as in Fig. S8. The video also shows additional mature *D. grandis*, dividing protoplast, and motile, non-loricate cells. Total length of video corresponds to 100 min.

Supplementary Video S2. *Diaphanoeca grandis*. Mature protoplast abandoning its lorica 3.8 days after the culture had been inoculated into the oCelloScope. Same parental cell indicated by arrow as in Fig. S11. The renewed lorica, also shown in Fig. S11 is also indicated by arrow. The video also shows additional mature *D. grandis*, dividing protoplast, motile, non-loricate cells, and cells forming a lorica. Total length of video corresponds to 100 min.
